# Profilin 1 as a Target for Cathepsin X Activity in Tumor Cells

**DOI:** 10.1371/journal.pone.0053918

**Published:** 2013-01-10

**Authors:** Urša Pečar Fonović, Zala Jevnikar, Matija Rojnik, Bojan Doljak, Marko Fonović, Polona Jamnik, Janko Kos

**Affiliations:** 1 Faculty of Pharmacy, University of Ljubljana, Ljubljana, Slovenia; 2 Department of Biochemistry, Molecular and Structural Biology, Jozef Stefan Institute and Centre of Excellence for Integrated Approaches in Chemistry and Biology of Proteins, Ljubljana, Slovenia; 3 Biotechnical Faculty, Ljubljana, Slovenia; 4 Department of Biotechnology, Jozef Stefan Institute, Ljubljana, Slovenia; Glasgow University, United Kingdom

## Abstract

Cathepsin X has been reported to be a tumor promotion factor in various types of cancer; however, the molecular mechanisms linking its activity with malignant processes are not understood. Here we present profilin 1, a known tumor suppressor, as a target for cathepsin X carboxypeptidase activity in prostate cancer PC-3 cells. Profilin 1 co-localizes strongly with cathepsin X intracellularly in the perinuclear area as well as at the plasma membrane. Selective cleavage of C-terminal amino acids was demonstrated on a synthetic octapeptide representing the profilin C-terminal region, and on recombinant profilin 1. Further, intact profilin 1 binds its poly-L-proline ligand clathrin significantly better than it does the truncated one, as shown using cathepsin X specific inhibitor AMS-36 and immunoprecipitation of the profilin 1/clathrin complex. Moreover, the polymerization of actin, which depends also on the binding of poly-L-proline ligands to profilin 1, was promoted by AMS-36 treatment of cells and by siRNA cathepsin X silencing. Our results demonstrate that increased adhesion, migration and invasiveness of tumor cells depend on the inactivation of the tumor suppressive function of profilin 1 by cathepsin X. The latter is thus designated as a target for development of new antitumor strategies.

## Introduction

Cancer is the second greatest cause of death in the developed world. To improve prevention, diagnosis and treatment, it is necessary to understand the molecular mechanisms of tumor development and progression in order that targets for the development of effective drugs and diagnostic tools can be identified. A number of molecules have been suggested to promote malignant processes, among them being cysteine cathepsins, such as cathepsin X [1], [2].

Cathepsin X is up-regulated in prostatic intraepithelial neoplasia and prostate cancer [2], [3] and suggested to be involved in the early stages of tumor development [2]. Cathepsin X is also up-regulated in gastric cancer [4] and hepatocellular carcinoma [5]. In the latter it may induce an epithelial to mesenchymal transition, an important process promoting tumor metastasis and malignancy by increasing cell motility and decreasing cell-cell adhesion [5].

The mechanism that links cathepsin X carboxypeptidase activity with the progression of cancer is not known. In contrast to cathepsin B, which promotes tumor invasion and metastasis by degrading proteins of the extracellular matrix, cathepsin X acts solely as a carboxypeptidase. However, as shown in the PymT-induced breast cancer mouse model of singly and doubly deficient cathB^−/−^cathX^−/−^ mice [6], [7], cathepsin X is able to promote tumor growth and invasion, and only silencing of the activity of both cathepsins significantly impairs tumor progression. Molecular targets other than the extracellular matrix have been identified which may be involved in the tumor promotion function of cathepsin X. The pro-peptide of cathepsin X posesses an RGD motif that binds to integrins, in particular α_ν_β_3_, thus mediating adhesion and migration of tumor cells [8]. Other molecular targets have been proposed as substrates for cathepsin X carboxypeptidase activity, cleaving the functional C terminal of the molecule: CXCL-12 chemokine [9] and beta-2 chain of the integrin receptor [10], [11], both influencing cell motility, adhesion, proliferation and migration of immune cells, and gamma-enolase, a glycolytic enzyme acting as a growth factor in neuronal cells [12] and used as a marker for prognosis and response to therapy in lung cancer and neuroblastoma.

The aim of the present study was to identify targets for cathepsin X carboxypeptidase activity in cancer cells. Profilin 1, a known tumor supressor factor, was identified as a candidate and cathepsin X was shown to be able to cleave its C-terminal and regulate its cellular function.

## Materials and Methods

Matrigel and fibronectin were from Becton Dickinson; all secondary antibodies, conjugated with Alexa Fluor were from Invitrogen; control siRNA, goat anti β_2_-integrin, goat anti α-enolase and goat anti γ-enolase antibodies were from Santa Cruz Biotechnology; anti-profilin 1 (C-terminal) antibody and mouse anti β-actin antibody were from Sigma; goat polyclonal anti-cathepsin X antibody, recognizing pro- and mature forms, was from R&D Systems; mouse monoclonal (X22) anti-clathrin antibody was from Abcam; anti-rabbit HRP and anti-mouse HRP antibodies were from Millipore.

Recombinant cathepsin X was prepared in *Pichia pastoris* [13]. Cathepsin X substrate Abz-FEK(DNP)OH was synthesized by Jiangsu Vcare Pharmatech Co. (China). Epoxysuccinyl-based cathepsin X inhibitor AMS-36 was synthesized as reported previously [14]. It was shown to specifically inhibit cathepsin X in tumor tissue [14], [15].

### Cell Culture and Transfection

Human prostate cancer cells (PC-3) were from ATCC, cultured in Advanced DMEM (Gibco) and F-12 (1∶1) with 10% FBS, 1% L-glutamine and 1% penicillin/streptomycin. Cathepsin X was transiently silenced using Lipofectamine 2000 (Invitrogen) and cathepsin X specific small interfering RNA (Invitrogen) according to the Instructions Manual.

### Preparation of Cell Lysates

For activity studies and immunoprecipitation analysis cells were treated with cathepsin X inhibitor AMS-36 (10 µM) or DMSO (0.1%) as a control for 24 hrs. Cell lysates were prepared in lysis buffer (50 mM HEPES pH 5.5, 1 mM EDTA, 150 mM NaCl, 1% Triton X-100) with protease inhibitor cocktail (Thermo Scientific) added. Total protein concentration was determined by DC Protein Assay (Bio Rad) according to instructions.

### Determination of Cathepsin X Activity

Cathepsin X activity was determined using the cathepsin X specific fluorogenic substrate Abz-Phe-Glu-Lys (Dnp)-OH [16]. The substrate is not hydrolyzed by related cathepsins L and B. 5 µM substrate in lysate sample and assay buffer (0.2 mg/ml final protein concentration in 100 mM acetate buffer, pH 5.5 containing 0.1% (w/v) polyethylene glycol 8000, 5 mM cysteine and 1.5 mM EDTA) were added to the wells of a black microplate. Formation of fluorescent degradation products was monitored continuously at 320 nm±5 nm excitation and 420 nm±5 nm emission on a Tecan Safire^2^™ spectrofluorimeter (Tecan Group Ltd).

### Quantitative ELISA

Microtiter plates were coated overnight with polyclonal anti-cathepsin X antibody (R&D Systems) in carbonate/bicarbonate buffer, pH 9.4 at 4°C (2.5 µg/ml; 50 µl/well). After washing with phosphate buffer saline with 0.05% Tween (PBST) and blocking with 2% BSA in PBST, samples or cathepsin X standard solutions were added (100 µl/well). Following a 2 hour incubation at 37°C, the wells were washed and filled with monoclonal 3B10-HRP conjugate, prepared by our group (1∶2500 dilution; 100 µl/well) [17]. Wells were washed and filled with 3,3′,5,5′-tetramethylbenzidine (TMB) liquid substrate system (Sigma) containing H_2_O_2_. The reaction was stopped after 15 min by adding 50 µl of 2 M H_2_SO_4_. The absorbance was measured at 450 nm and the concentration of cathepsin X calculated from the calibration curve.

### Real Time Cell Migration, Invasion and Adhesion Assays

Cell assays were done on a Real-Time Cell Analyzer Dual Plate (RTCA DP) Instrument, xCELLigence System (Roche Applied Science) [18]. This novel technology is based on real-time monitoring of cell invasion, migration or adhesion and it captures cell responses during the entire course of an experiment, otherwise missed if measured by conventional endpoint assays. For migration, CIM plates (cell invasion and migration) were coated with fibronectin (10 µg/ml) on the down- and upper-sides of the microporous PET membrane for 30 min at room temperature and 2 hrs at 37°C, respectively. Excess fibronectin was removed and wells washed with phosphate buffer saline (PBS). Lower chambers were filled with complete medium containing cathepsin X inhibitor (10 µM) or DMSO. Upper chambers were filled with serum-free medium with cathepsin X inhibitor (10 µM) or DMSO. 2×10^4^ cells were plated per well.

For invasion, only the down-side of the membrane was coated as for the migration assay, whereas the upper-side was coated with 50% Matrigel (20 µl) in serum-free medium for 30 min at 37°C. 3×10^4^ cells were plated per well.

For adhesion, E plates were coated with fibronectin (10 µg/ml) for 1 hour at 37°C and washed with PBS. 5×10^3^ cells were plated per well in complete medium with cathepsin X inhibitor (10 µM) or DMSO.

The Cell Index (CI) represents the relative change in electrical impedance to indicate cell status. Dynamic CI values were monitored at 15 min intervals from the time of plating until the end of the experiment (72 h). Data were analyzed with the RTCA Software.

### Two-dimensional Electrophoresis and Protein Identification

PC-3 cells were treated with 10 µM cathepsin X inhibitor, or DMSO as a control, and harvested after 24 h. Cells were then solubilized in lysis buffer [2 M thiourea (Sigma), 7 M urea (Sigma), 4% CHAPS (Sigma), 1% dithiothreitol (Sigma), 2% IPG buffer (GE Healthcare) and 1×inhibitor cocktail tablet (Roche)]. Samples (containing 200 µg of total protein) were mixed with rehydration solution [2 M thiourea, 7 M urea, 2% CHAPS, 2% IPG buffer and 0.002% of bromophenol blue] and separated by isoelectric focusing using 13-cm immobilized pH 3–10 non-linear gradient (IPG) strips (GE Healthcare) according to instructions. After the first dimension, IEF strips were transferred separately to an equilibration solution (6 M urea, 75 mM Tris base, 2% [w/v] SDS, 30% glycerol [v/v], 0.002% bromophenol blue [w/v], 65 mM DTT) and incubated for 15 minutes at room temperature with shaking. Strips were then transferred to the same solution, but without DTT and with added iodoacetic acid (260 mM), and incubated for 15 minutes at room temperature with shaking. Strips were transferred horizontally onto 12% [w/v] polyacrylamide gels and covered with 1.2% [w/v] agarose. Second dimension gels were run at 50 mA/gel for 6 h. Proteins were visualized by SYPRO Ruby Protein Stain (BioRad), followed by in-gel digestion of the selected protein spots by trypsin. Peptides were analyzed with a 1200 Series HPLC System coupled to an MSD Trap XCT Ultra mass spectrometer using a protein identification chip (all Agilent Technologies). Conditions were the following: 40 nl enrichment column (Zorbax 300 SB C18), 75 µm×150 mm analytical column (same packing); mobile phase: 0.1% formic acid and acetonitrile; gradient: 0–41 min, 3–50%. The three most intense peaks in each scan cycle were chosen for CID fragmentation. Dynamic exclusion was enabled at count 2 and exclusion duration 30 sec. Data was analyzed with Spectrum Mill MS Proteomics Workbench software (Agilent Technologies) and searched against the NCBInr database. Carbamidomethylation of cysteins was set as a fixed modification and oxidation of methionines was set as a variable modification.

### Co-localization Studies

5×10^2^ cells/ml were plated on cover slips. Cells were fixed with 10% formalin (Sigma-Aldrich), permeabilized with 0.025% Triton X-100 (Serva) and incubated with primary antibodies for 2 hours. After washing with PBS they were incubated for 2 hours with Alexa Fluor labeled secondary antibodies. Prolong Antifade kit (Molecular Probes) was used for mounting the coverslips on glass slides. Fluorescence microscopy was performed using a Zeiss LSM510 confocal microscope with LSM image software, version 3.0 or a Zeiss LSM 710 confocal microscope with ZEN 2011 image software.

### Digestion Studies

A synthetic octapeptide S^132^ - Y^139^ corresponding to the 8 C-terminal amino acids of profilin 1, was synthesized by EZBiolab, USA. Digestion with recombinant cathepsin X (4.62 µM) was performed at 37°C for 30 minutes in 100 mM acetate buffer (pH 5.5) with 5 mM cysteine and 1.5 mM EDTA. The product was separated by reverse-phase HPLC using a C18 Gemini column (5 µm, 110 Å, 150×4.6 mm) (Phenomenex). Peaks were analyzed by a Q-TOF Premier mass spectrometer (in ESI+ mode).

1 µg/µl of profilin 1 (Abcam) was digested with cathepsin X (46.2 µM) at 37°C. Aliquots were taken at different time points (0 min, 1 h, 2 h, 4 h and 8 h) and the reaction stopped with a change of pH, from 5.5 to 3.0, with TFA and subsequent freezing at −80°C. Samples were analyzed using Ultraflextreme Maldi TOF/TOF.

### Flow Cytometry Analysis

For the actin polymerization study, a 6-well plate was coated overnight with fibronectin (10 µg/ml) at 4°C. 2×10^5^ cells/well were plated and, after 24 hrs 10 µM AMS-36 or DMSO was added for 24 hrs. Cells were trypsinized, fixed with 10% formalin for 10 min on ice and permeabilized with 0.1% Triton X-100 in PBS for 15 min at room temperature in the dark. Cells were resuspended in PBS and incubated with phalloidin-tetramethylrhodamine B isothiocyanate conjugate (Sigma) for 1 h on ice in the dark. After washing twice with PBS, the fluorescence signal was analyzed on a FACSCalibur flow cytometer with Cell Quest software.

### Immunoprecipitation and Western Blotting

Protein A Sepharose beads (GE Healthcare) were washed twice with lysis buffer and added to the cell lysate (1.8 mg total protein; buffer : lysate was 1∶1 v/v). The mixture was incubated for 30 min at 4°C with shaking and centrifuged for 3 min (4°C). Anti-clathrin antibody (1 µg) or anti β-actin antibody (5 µg) was added to the supernatant and incubated overnight at 4°C with constant shaking. Washed Sepharose beads were added and incubated for 3 hrs at 4°C. The sample was diluted with 2 volumes of lysis buffer, centrifuged for 5 min and immune complexes eluted from the beads by boiling for 10 min in SDS sample buffer. Proteins were separated on 15% glycine gels and transferred to a Hybond-N nitrocellulose membrane (GE Healthcare). Membranes were blocked with 5% skimmed milk powder in PBS for 1 hour, incubated with primary antibodies in PBST for 1 hour and finally with secondary antibodies in PBST for 45 min. Proteins were detected with SuperSignal West Dura Extended Duration Substrate chemiluminescence kit (Thermo Scientific).

## Results

### Cathepsin X Activity and Protein Levels in Cell Lysates

**Figure 1:**
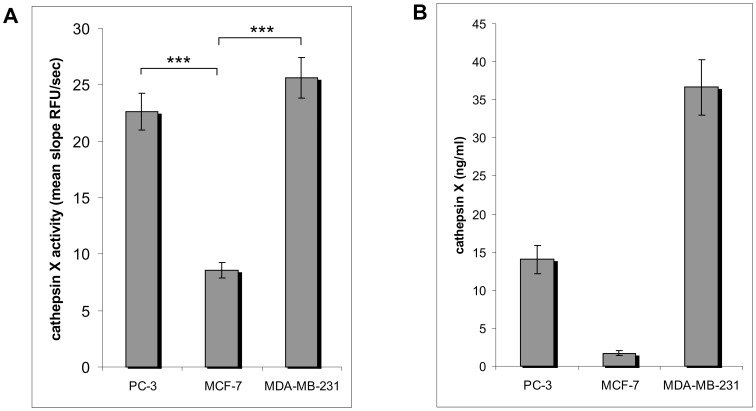
Cathepsin X in MCF-7, MDA-MB-231 and PC-3 cells. (**A**) Activity was measured in cell lysates using cathepsin X specific substrate Abz-FEK(Dnp)-OH. Mean values of 5 independent experiments are shown. ***P<0,001 (**B**) The amount of cathepsin X (ng/ml) in different cell lines was determined with ELISA. Mean values of 2 (MCF-7 and MDA-MB-231) or 4 (PC-3) independent experiments are shown.

Carboxypeptidase activity of cathepsin X was determined in three cancer cell lines, prostate cancer PC-3, the highly invasive breast cancer MDA-MB-231 [19] and the less invasive breast cancer MCF-7 [19], using Abz-Phe-Glu-Lys (Dnp)-OH as substrate. In PC-3 and MDA-MB-231 cells the levels of enzyme activity and enzyme protein were significantly higher than in MCF-7 cells (Fig. 1A and 1B). We selected PC-3 cells for the further experiments due to the highest specific activity of cathepsin X.

### Cathepsin X Increases Migration, Adhesion and Invasion of PC-3 Cells

**Figure 2:**
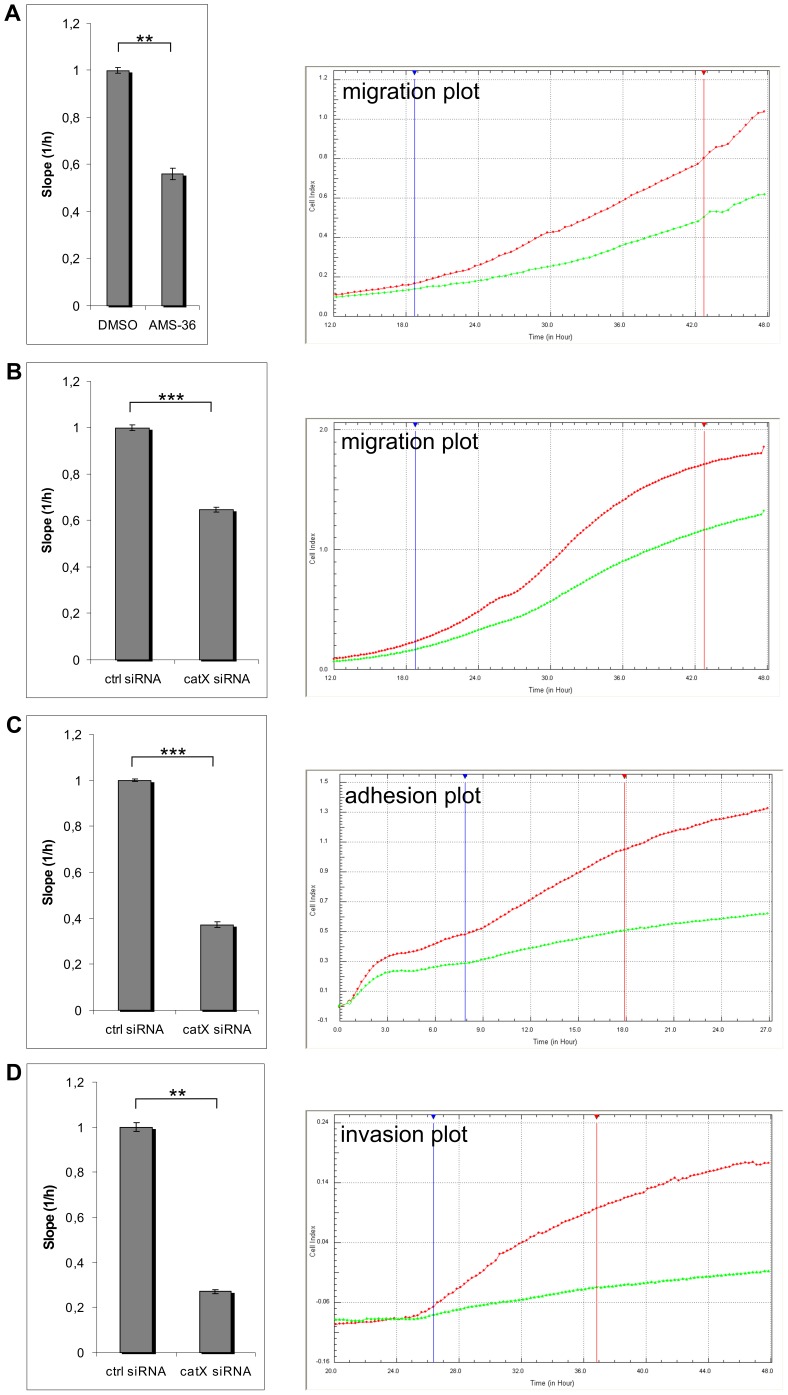
Cathepsin X increases migration, adhesion and invasion of cancer cells. Migration (A, B), adhesion (C) and invasion (D) assays were done using xCELLigence System. (**A and B**) Diagrams show a slope (cell index vs. time) of cells that migrated from the upper to the lower chamber. Cells migrated in the presence of DMSO (red line on graph) or 10 µM inhibitor of cathepsin X (green line on graph) (A) or cells, transfected with control (red line on graph) or cathepsin X specific siRNA (green line on graph) were used (B). (**C**) Diagram shows a slope for cells transfected with a control (red line on graph) or cathepsin X specific siRNA (green line on graph), that adhered to fibronectin (10 µg/ml). (**D**) Diagram shows a slope for cells transfected with a control (red line on graph) or cathepsin X specific siRNA (green line on graph) that invaded through Matrigel from the upper to the lower chamber. ***P≤0.01; ***P<0,001. Graphs show real-time curves of cell index (CI) as a function of time. Vertical lines represent the start and end of time intervals within which corresponding diagrams are calculated. Four (A), eight (B), four (C) and five (D) biological repeats were performed.

As shown by real time analysis the cathepsin X inhibitor AMS 36 decreased migration of PC-3 cells across fibronectin by 42% (Fig. 2A, green line). To confirm the impact of cathepsin X on cell migration we transiently silenced cathepsin X in PC-3 cells with siRNA (Fig. S1). The result was similar, the migration being decreased by 38% (Fig. 2B, green line). The adhesion of PC-3 cells on a fibronectin-coated surface with transiently silenced cathepsin X was 63% lower (Fig. 2C, green line) than that of those transfected with control siRNA. Further, the invasion of silenced cathepsin X cells over a thick layer of Matrigel was 77% lower than that of the control (Fig. 2D, green line).

### Profilin 1 is a Candidate for Cathepsin X Substrate

**Figure 3:**
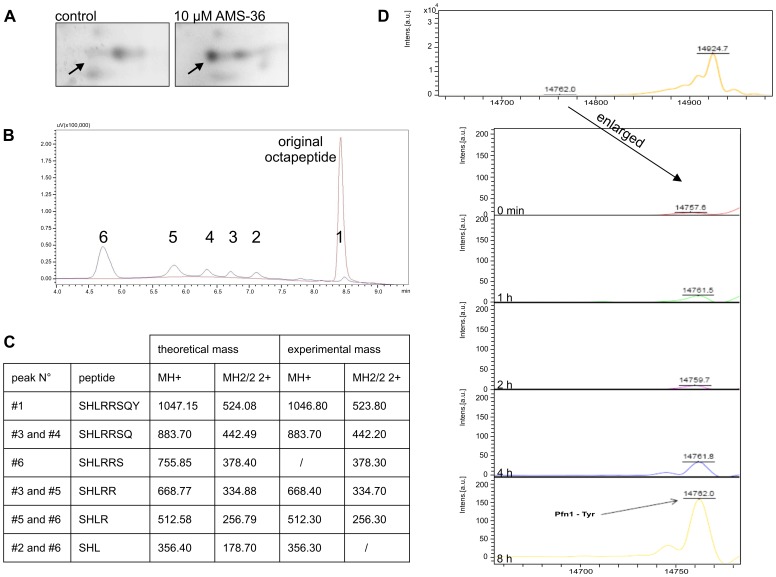
Identification of profilin 1 as a substrate for cathepsin X carboxypeptidase activity. (**A**) Control versus AMS-36 treated sample is shown after 2D electrophoresis. The spot marked with arrow was identified as human profilin 1. (**B and C**) The C-terminal of profilin 1 (SHLRRSQY) (800 µM) was digested with recombinant cathepsin X (4.62 µM) at 37°C for 30 minutes and separated on a C18 Gemini column (5 µm, 110 Å, 150×4.6 mm) (Phenomenex). (**B**) 5 additional peaks, named peaks 2 to 6, were detected besides the original octapeptide (black line). The octapeptide control without enzyme is shown in red. (**C**) Q-TOF Premier mass spectrometry analysis of each peak showed the presence of 3 to 7 amino acid long peptides, all shortened by 1 amino acid from the C- terminal. (**D**) Profilin 1 (1 µg/µl; Abcam) was digested with recombinant cathepsin X (46.2 µM) at 37°C for several hours and the digestion product detected with mass spectrometry. A new peak was detected with molecular mass matching the mass of profilin 1 without the last amino acid residue Tyr.

PC-3 cells were treated with AMS-36 for 24 h and lysates prepared for 2D electrophoresis. After protein detection with SYPRO Ruby Protein Stain, spots showing different intensities in treated and non-treated samples were chosen for further investigation on mass spectrometry. A spot corresponding to around 16 kDa, with intensity 1 in non-treated cells and intensity 3.4 (Fig. 3A, arrow) in treated cells, was identified as human profilin 1, a protein known to influence the motility of invasive cancer cells [20].

A synthetic octapeptide that represents the C-terminal of profilin 1 (SHLRRSQY) was cleaved with cathepsin X *in vitro*. After 30 minutes digestion at 37°C several shorter peptides were detected on reverse-phase HPLC (Fig. 3B). Mass spectrometry analysis showed the presence of 3 to 7 amino acid long peptides (Fig. 3C), successively shortened by one amino acid from the C-terminal part. No peptides shortened at the N-terminal were observed.

Incubation of cathepsin X with the recombinant profilin 1 resulted in cleavage of the last amino acid Tyr^139^, as detected with mass spectrometry (Fig. 3D).

### Cathepsin X and Profilin 1 Co-localize with Actin and Clathrin

**Figure 4:**
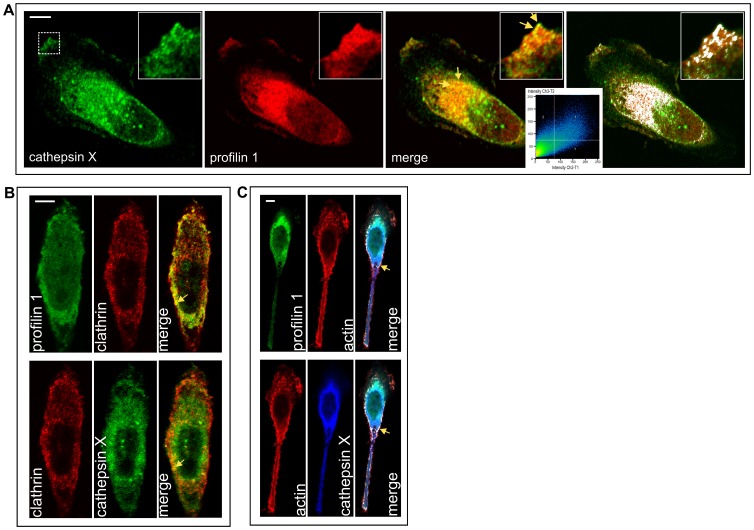
Co-localization of cathepsin X and profilin 1 (A) with clathrin (B) and actin (C). All proteins were visualized by immunofluorescence staining using primary antibodies to cathepsin X, profilin 1 and clathrin, followed by Alexa Fluor conjugated secondary antibodies Alexa Fluor 488, 555 and 633 or phalloidin conjugate for actin. (**A**) Cathepsin X is shown in green, profilin 1 in red and co-localization in yellow. For co-localization, also Zen 2011 Software (Carl Zeiss) option for improved visibility of co-localized pixels was used and co-localization is shown in white with corresponding scatter diagram. (**B**) Clathrin is shown in red, profilin 1 and cathepsin X are both in green due to clearer merged image. (**C**) Profilin 1 is shown in green, cathepsin X in blue and actin in red. Zen 2011 Software option for improved visibility of co-localization is used. Bars, 10 µm.

Profilin 1 is a cytosolic protein, however it is often bound to the plasma membrane (plasmalemmal or intracellular) and is present also in the nucleus. Profilin 1 and cathepsin X staining showed a strong co-localization on the plasma membrane as well as in the perinuclear region (Fig. 4A, arrows). Triple co-localization with two known binding partners of profilin 1 clathrin (Fig. 4B) and actin (Fig. 4C) showed both to be co-localized with profilin 1 and cathepsin X in a perimembrane area (arrows). No co-localization was observed with targets previously reported for other cells (Fig. S2).

### Interaction of Profilin 1 and Clathrin is Cathepsin X Dependent

**Figure 5:**
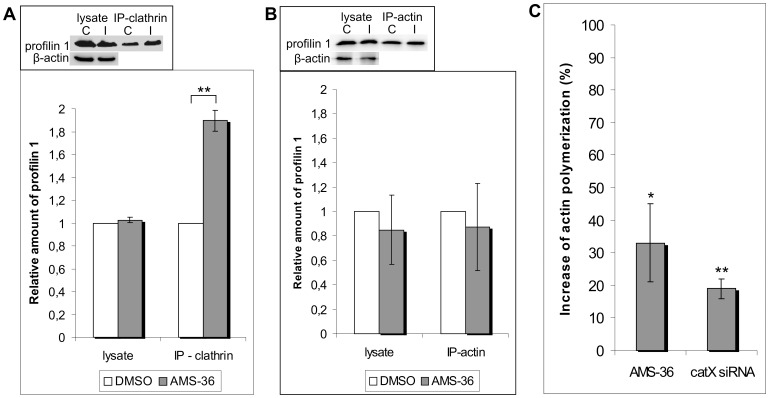
Cathepsin X modulates profilin 1 function by affecting the binding of poly-L-proline ligands. (**A and B**) Profilin 1 forms a stable complex with actin and clathrin. Representative co-immunoprecipitations of profilin 1 with clathrin (A-inset) and actin (B-inset) in PC-3 cells, treated with DMSO or cathepsin X specific inhibitor. (**A and B insets**) Cell lysates were treated with anti-clathrin or anti β-actin antibody and immunoprecipitated on Protein A Sepharose beads. Profilin 1 was detected by Western blot in total cell lysates and in immunoprecipitated pellets. β-actin was used as a loading control. (**A and B**) Quantification of data from the insets. The graphs represent densitometric analysis of bands using Sygene's GeneTools Software (Sygene, U.K.). Three or two biological experiments indicate the normalized amount of profilin 1 that is in complex with clathrin (A) or actin (B), respectively. **P≤0.01. (**C**) Cathepsin X regulates actin polymerization. Flow cytometric analysis of permeabilized PC-3 cells is shown. Filamentous actin was stained with phalloidin conjugate. Cells were treated with DMSO or AMS-36 or were transfected with control or cathepsin X specific siRNA. Increase in actin polymerization is shown with values of both control experiments set to 0% increase in actin polymerization. Values are representative of four independent experiments. *P≤0.05; **P<0.01.

Clathrin is a poly-L-proline ligand of profilin 1, binding to the C-terminal of the profilin molecule. To confirm their interaction, proposed by co-localization, their assumed complex was immunoprecipitated with anti-clathrin antibody and Protein A Sepharose beads. The bound proteins were separated on SDS-PAGE and profilin 1 detected on Western blot (Fig. 5A, inset). There was almost 44% more profilin 1 complexed with clathrin when cells were treated with AMS 36 inhibitor (Fig. 5A), showing that profilin 1, when digested with cathepsin X at the C-terminal, is less prone to bind clathrin. Additionally, we immunoprecipitated profilin 1 and actin. The actin binding site is at the opposite site of the profilin 1 molecule and should not depend on cathepsin X action. As expected, the profilin 1/actin complex was unchanged, regardless of inhibitor treatment, as shown in Figure 5B and inset.

### Cathepsin X Regulates Actin Polymerization

The binding of certain poly-L-proline ligands enhances actin polymerization. We checked the level of filamentous actin in cells treated with AMS-36 and in those transfected with cathepsin X specific siRNA (Fig. 5C). Polymerization was higher by almost 30% when cathepsin X was inhibited and by 20% when cathepsin X was silenced. This additionally strengthens the importance of cathepsin X cleavage of profilin C-terminal for binding of ligands.

## Discussion

Cathepsin X degrades various molecular targets involved in signal transduction, growth, maturation, adhesion, cell-cell communication, proliferation and migration of immune and neuronal cells [9], [12], [21], [22]. Its important role was also suggested in tumor cells and results of our study clearly demonstrate the association of its carboxypeptidase activity with the invasiveness of prostate PC-3 cells and breast cancer MDA-MB-231 and MCF-7 cells. However, the molecular targets of cathepsin X, identified in other cell types, such as beta-2 chain of integrin receptors [23], and alpha- and gamma-enolase [12] were not confirmed as targets in tumor cells by co-localization studies (Fig. S2). Their co-localization and processing by cathepsin X seem to be specific for immune and neuronal cells and not for cancer cells. Here we report profilin 1 as a new target for cathepsin X, which is co-localized with the enzyme in PC-3 cells. Its processing at the C terminal may significantly affect its tumor suppressive function and promotes cell migration and invasion as demonstrated by using specific cathepsin X inhibitor and siRNA silencing.

Profilin 1 is an important protein in tumorigenesis and was discovered as a protein sequestering actin monomers [24]. Later it was found to be a promoter of actin polymerization [25]. Higher levels of profilin 1 act to inhibit tumor progression, and its down-regulation has been reported in different types of adenocarcinoma (breast, hepatic, pancreatic) [26]. Profilin 1 is a 15 kDa protein with three known binding sites: poly-L-proline ligands bind to the cleft between C- and N- terminal helices; actin and actin related proteins (ARPs) and gephyrin bind on the opposite site of the profilin molecule, and the binding site for phosphatidylinositol lipids (PI) overlaps those for actin and poly-L-proline [27].

The C-terminal of profilin is part of the binding site for 2 important groups of ligands: (1) poly-L-proline ligands, including membrane trafficking proteins (like clathrin, huntingtin), members of Rac/rho or cdc42 signaling, synaptic scaffold proteins, and builders of focal contacts (reviewed in 25) and (2) phosphoinositides, known to inhibit motility of MDA-MB-231 cells when associated with profilin 1 [28].

The poly-L-proline binding site consists of highly conserved aromatic and hydrophobic amino acid residues: Lys124, His133 and Tyr139 on the C-terminal helix; Trp3, Tyr6 and Trp31 on the N-terminal helix [29] and the later discovered domain with Tyr 66, Tyr72 and Tyr106 [30]. The C-terminal region is also very important for the stability of profilin 1.

The mechanisms by which profilin 1 achieves specificity in the plethora of poly-L-proline ligands are not known. A possible explanation is a phosphorylation of certain amino acid residues. Phosphorylation of Ser137 and Tyr139 interferes with poly-L-proline binding [29] whereas the phosphorylation of tyrosines in the N- and C- terminal domains and on position 72 regulates binding of PI3K in bean [30].

Since the C-terminal of profilin 1 protrudes from the otherwise compact structure [31], it could be a potencial site of proteolytic digestion by cathepsin X carboxypeptidase activity. We have identified profilin 1 as a possible target for cathepsin X by differential analysis of control cells and those treated with cathepsin X inhibitor AMS 36, using 2D electrophoresis and mass spectrometry. The C-terminal cleavage of profilin 1 by cathepsin X was confirmed on the octapeptide SHLRRSQY corresponding to the C-terminal of profilin 1. Several cleaved products were obtained, ranging from 7 to 3 amino acids, the expected result of cathepsin X monocarboxypeptidase activity. As a control, two peptides, whose sequences differed from that of profilin 1 were treated with cathepsin X (Fig. S3). The lack of cleavage products confirmed the specific action of cathepsin X on profilin 1 C-terminal amino acid sequence. When the intact, recombinant profilin 1 was treated with cathepsin X, cleavage of the last C-terminal amino acid residue, Tyr139, was identified. As noted above, Tyr139 is one of the regulating residues that can be phosphorylated, and its cleavage may affect the binding of poly-L-proline ligands and the function of profilin 1. The difference in cleavage specificity and efficiency between the octapeptide and recombinant profilin 1 can be explained by different tertiary structure in both molecules. Also, the native profilin 1 may differ from the recombinant one regarding the accessibility of the C terminus, which depends on the stability of other parts of the molecule, in particular N terminal helix [29]. Further studies are needed to solve the precise mode of action of cathepsin X on profilin 1 in living cell.

Profilin 1 and cathepsin X co-localize strongly at the plasma membrane and in the perinuclear area. Clathrin and actin, two important profilin 1 binding proteins, showed co-localization with cathepsin X and profilin 1, in particular in the area close to the plasma membrane. When we co-immunoprecipitated profilin 1 and clathrin in the presence and absence of AMS-36, the cells treated with the inhibitor exhibited 40% more profilin 1 in complex with clathrin than non-treated cells. Clearly, the cleavage of profilin 1 at the C-terminal weakened its association with clathrin. Aberrant recycling and vesicular trafficking is one of the aspects of malignant cells [32] and by preventing firm profilin 1/clathrin complexes cathepsin X may cause changes in clathrin dependent endocytosis.

On the other hand cathepsin X inhibition had no effect on the binding of profilin 1 and actin, suggesting that the actin binding site is not affected by the cleavage of profilin 1. Actin cytoskeleton reorganization is the main regulatory mechanism of cancer cell migration which is an important step of tumor invasion [33]. Action of profilin 1 on actin cytoskeleton has a very complex nature, depending on parameters which vary between different cell types as is discussed in Ref. 20. Profilin 1 may either sequester G-actin and by that inhibit actin polymerization or promote actin assembly [34]. As tumor suppressor, profilin 1 was reported many times to inhibit cell motility and matrigel invasivness [20], [28], [35], but exact mechanism has not been discovered yet. We showed that PC-3 cells with inhibited cathepsin X had higher F-actin content (filamentous actin) than cells with active cathepsin X. In literature, for profilin 1-specific siRNA treated MDA-MB-231 cells, reduced F-actin content and reduced F-actin staining near the leading edge were shown [20], whereas overexpressed or microinjected profilin 1 increased overall F-actin [36]. We may hypothesize that by cleaving C-terminal of profilin 1, cathepsin X regulates actin polymerization and consequently cell migration. The cleavage of profilin 1 may interfere also binding to multidomain proteins like Arp2/3 complex, WASP and VASP which are poly-L-proline rich ligands with binding sites for profilin 1 and actin (in globular and filamentous form). They act as recruiters of the profilin 1/actin complexes to the sites of filamentous actin elongation near the plasma membrane [37]. The cleavage of profilin 1 may also interfere the binding to phosphoinositides, another important molecules regulating cell motility [28].

In conclusion, profilin 1 has been identified as an important molecular target of cathepsin X in tumor cells. Since profilin 1 acts as a tumor suppressor, the impairment of its function may lead to increased motility and invasion of tumor cells, as shown in this and other studies [20]. However, our results also suggest the possible application of cathepsin X inhibitors in the treatment of malignant diseases.

## Supporting Information

Figure S1 **Cathepsin X silencing in PC-3 cells.** PC-3 cells were transfected with control or cathepsin X specific siRNA using Lipofectamine. After 24, 48 and 72 hours, cell lysates were prepared and cathepsin X activity measured using Abz-FEK(Dnp)-OH substrate (**A**) and the amount of cathepsin X (ng/ml) determined with ELISA (**B**). Mean values of three (control and 24 h) or two (48 h and 72 h) separate experiments are shown. *P<0.05; **P≤0.01 (**C**) Representative image of Western blot of the lysates of cells silenced for cathepsin X using anti-cathepsin X antibody.
(TIF)

Figure S2 **Co-localization of cathepsin X with α-enolase, γ-enolase and β_2_-integrin in PC-3 cells.** All proteins were visualized by immunofluorescence staining using antibodies to cathepsin X, α-enolase, γ-enolase or β_2_-integrin, followed by Alexa Fluor conjugated secondary antibodies, Alexa Fluor 488 (green) for cathepsin X and Alexa Fluor 555 (red) for α-enolase, γ-enolase or β_2_-integrin. Bars, 5 µm.
(TIF)

Figure S3 **Cathepsin X action on control octapeptides.** Octapeptides LFPITSVL (**A**) and AMEDASVL (**B**) (both 800 µM) were digested with recombinant cathepsin X (4.62 µM) at 37°C for 60 minutes and separated on a C18 Gemini column (5 µm, 110 Å, 150×4.6 mm) (Phenomenex).
(TIF)
