# Supplementary figures and images for: Profilin 1 as a Target for Cathepsin X Activity in Tumor Cells
Source: PLoS One. 2013 Jan 10;8(1):e53918. doi: 10.1371/journal.pone.0053918 (PMC3542269; doi:10.1371/journal.pone.0053918)

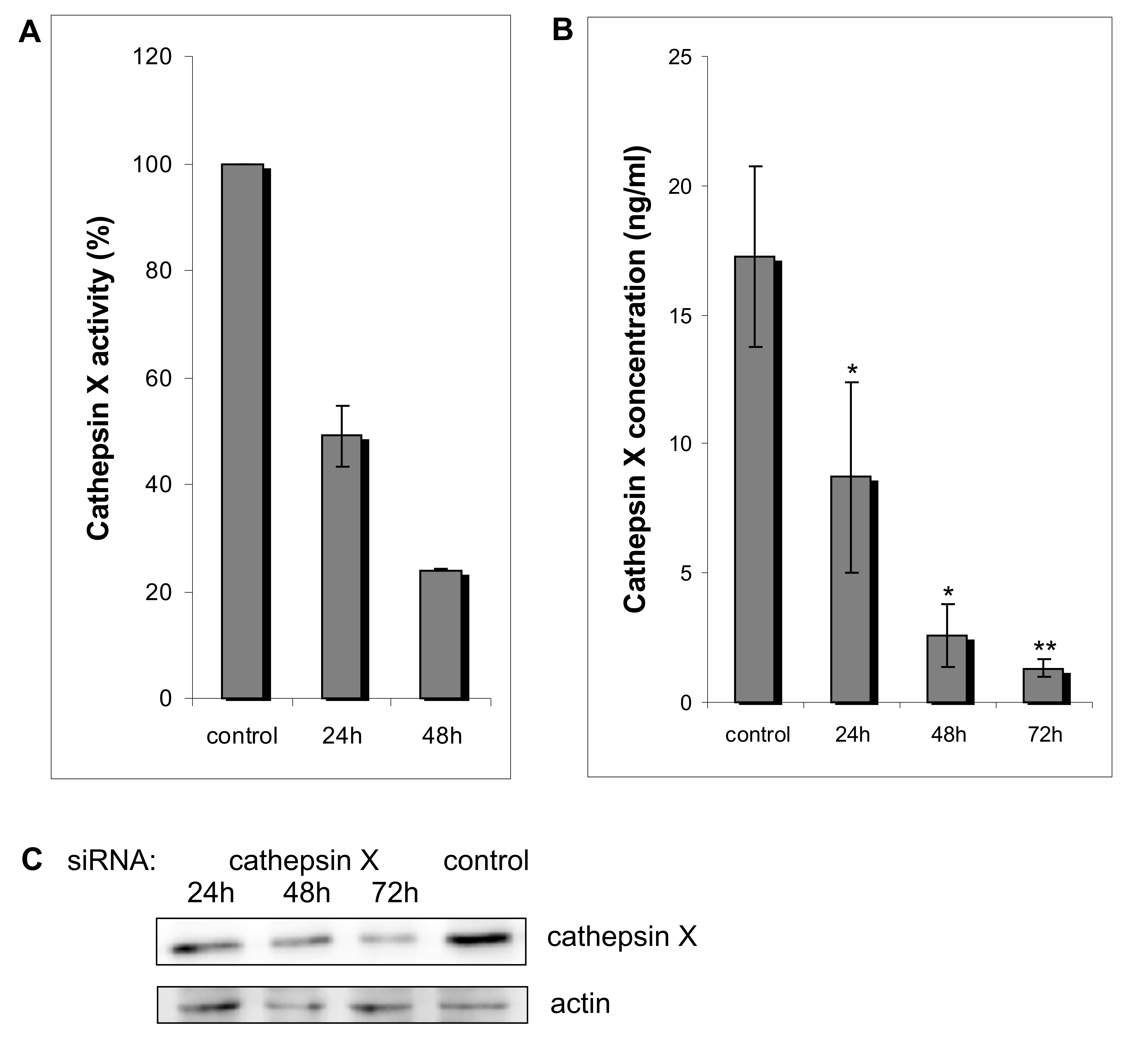

Supplement: Figure S1 — Cathepsin X silencing in PC-3 cells. PC-3 cells were transfected with control or cathepsin X specific siRNA using Lipofectamine. After 24, 48 and 72 hours, cell lysates were prepared and cathepsin X activity measured using Abz-FEK(Dnp)-OH substrate (A) and the amount of cathepsin X (ng/ml) determined with ELISA (B). Mean values of three (control and 24 h) or two (48 h and 72 h) separate experiments are shown. *P<0.05; **P≤0.01 (C) Representative image of Western blot of the lysates of cells silenced for cathepsin X using anti-cathepsin X antibody. (TIF) [file pone.0053918.s001.tif]

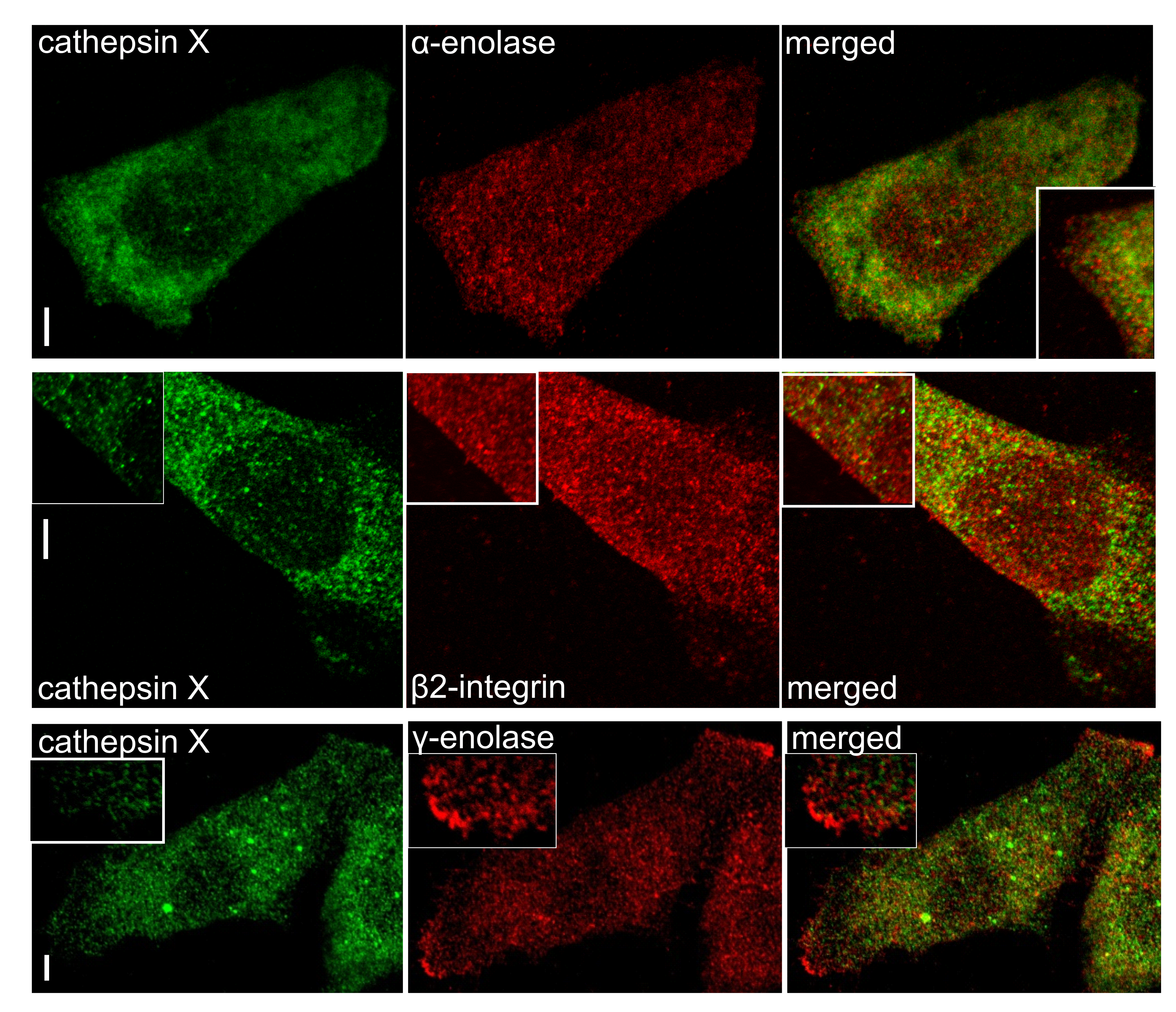

Supplement: Figure S2 — Co-localization of cathepsin X with α-enolase, γ-enolase and β2-integrin in PC-3 cells. All proteins were visualized by immunofluorescence staining using antibodies to cathepsin X, α-enolase, γ-enolase or β2-integrin, followed by Alexa Fluor conjugated secondary antibodies, Alexa Fluor 488 (green) for cathepsin X and Alexa Fluor 555 (red) for α-enolase, γ-enolase or β2-integrin. Bars, 5 µm. (TIF) [file pone.0053918.s002.tif]

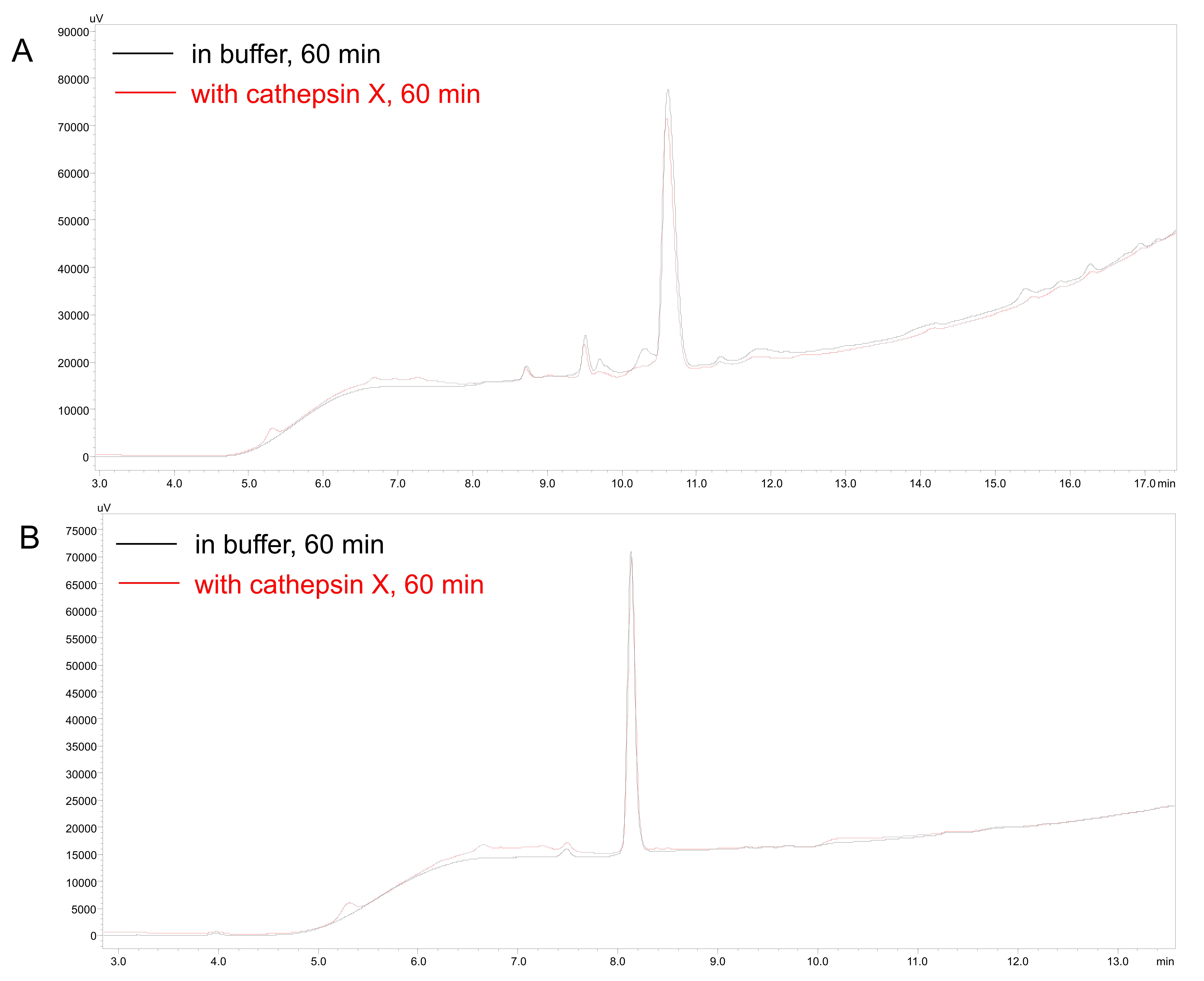

Supplement: Figure S3 — Cathepsin X action on control octapeptides. Octapeptides LFPITSVL (A) and AMEDASVL (B) (both 800 µM) were digested with recombinant cathepsin X (4.62 µM) at 37°C for 60 minutes and separated on a C18 Gemini column (5 µm, 110 Å, 150×4.6 mm) (Phenomenex). (TIF) [file pone.0053918.s003.tif]
